# Ultrafast Dynamics in Flavocytochrome C by Using Transient Absorption and Femtosecond Fluorescence Lifetime Spectroscopy

**DOI:** 10.1021/acs.jpcb.4c05496

**Published:** 2025-04-08

**Authors:** Krishna P. Khakurel, Gustavo Fuertes, Aron Sipos, Gábor Paragi, Jakub Dostal, Miroslav Kloz, Gabriel Žoldák, Jakob Andreasson, András Tóth

**Affiliations:** † Extreme Light Infrastructure ERIC, Dolni Brezany CZ-25241, Czech Republic; ‡ Institute of Biotechnology Czech Academy of Sciences, Vestec CZ-25250, Czech Republic; § Institute of Biophysics, HUN-REN Biological Research Centre, Szeged H-6726, Hungary; ∥ Institute of Physics, University of Pécs, Ifjúság útja 6, Pécs H-7624, Hungary; ⊥ Department of Theoretical Physics, University of Szeged, Tisza Lajos krt. 84-86, Szeged H-6720, Hungary; # Department of Medicinal Chemistry, University of Szeged, Dóm tér 8, Szeged H-6720, Hungary; ∇ Faculty of Science, Faculty of Science, Pavol Jozef Šafárik University in Košice, Park Angelinum 19, Košice 040 01, Slovakia; ○ Department of Biotechnology and Microbiology, University of Szeged, Szeged H-6726, Hungary

## Abstract

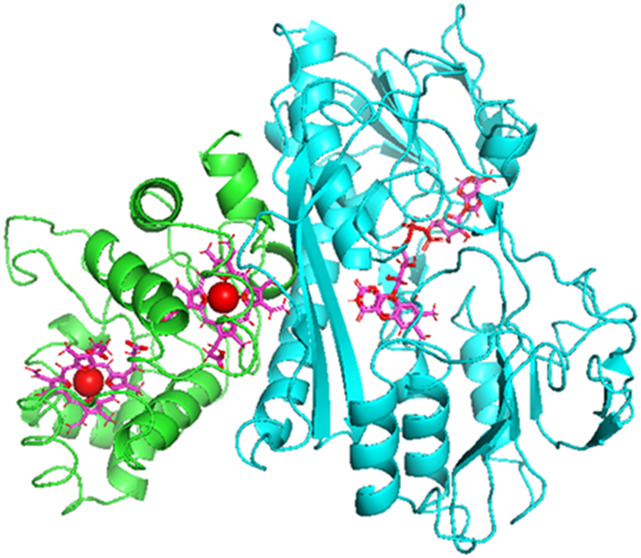

Flavocytochrome c sulfide dehydrogenase (FCC) is an important enzyme of sulfur metabolism in sulfur-oxidizing bacteria, and its catalytic properties have been extensively studied. However, the ultrafast dynamics of FCC is not well understood. We present ultrafast transient absorption and fluorescence spectroscopy measurements to unravel the early events upon excitation of the heme and flavin chromophores embedded in the flavocytochrome c (FccAB) from the bacterium *Thiocapsa roseopersicina*. The fluorescence kinetics of FccAB suggests that the majority of the photoexcited species decay nonradiatively within the first few picoseconds. Transient absorption spectroscopy supports these findings by suggesting two major dynamic processes in FccAB, internal conversion occurring in about 400 fs and the vibrational cooling occurring in about 4 ps, mostly affecting the heme moiety.

## Introduction

Among the sulfur species, sulfide is the most reduced and a very reactive one. Since sulfide is a highly toxic compound, primarily by inhibiting hemoproteins of electron transport chains, cells have developed sophisticated mechanisms to defend it.^1^ However, sulfide has also several physiological functions in living organisms. Chemotrophic and anaerobic phototrophic bacteria are able to utilize it as electron and energy source to the respiratory and photosynthetic chains, respectively.^2,3^ In higher eukaryotes, it is involved in important physiological processes as cell modulator and signaling molecule.^4,5^

The sulfide oxidase enzymes, which catalyze the oxidation of sulfide to elementary sulfur, play a basic role in sulfide detoxification, microbial energy conservation, and the regulation of cellular sulfide level. Two sulfide oxidase enzymes are known. The membrane-bound sulfide:quinone oxidoreductase (SQR) is widely distributed in prokaryotic and eukaryotic organisms, while the soluble periplasmic flavocytochrome c sulfide dehydrogenase (frequently named as flavocytochrome c, FCC) enzyme is present in chemolithotrophic sulfur-oxidizing bacteria^6^ and in phototrophic purple and green sulfur bacteria.^7,8^ Both of these enzymes are members of the two-Dinucleotide Binding Domains Flavoproteins (tDBDF) enzyme superfamily. According to the recent phylogenetic analysis of the sulfide oxidizing enzymes, the SQR proteins have four groups which consist of six classes, and the group of the FCC proteins can be divided into three subgroups.^9^ FCC is a heterodimeric enzyme that catalyzes the oxidation of sulfide to zero-valence sulfur, typically polysulfide, and transfers the released electrons to periplasmic small c-type cytochrome proteins. The big subunit of FCCs (FccB) is a FAD-binding flavoprotein which is an SQR-type protein. FccB and SQR proteins have a similar domain structure characteristic of tDBDF enzymes consisting of two Rossman-fold domains.

The first Rossman-fold is responsible for the FAD binding via noncovalent interactions. In FCC, the FAD is even bound covalently to a conserved cysteine residue by a thioether bond. The other Rossmann fold domain contains the residues forming the catalytic site for sulfide binding and oxidation. The third domain is the C-terminal domain, which is responsible for the binding of the electron acceptor of the reduced FAD cofactor. In the case of FCCs, the electron acceptor of the FAD is the small subunit (FccA) of the dimeric enzyme. FccA is a monohaem^10,11^ or a dihaem^12,13^ cytochrome c protein tightly associated with the FccB subunit by noncovalent interactions.

The structures of three FCC enzymes have been determined by X-ray crystallography from the purple sulfur bacteria *Allochromatium vinosum*(12) and *Thermochromatium tepidum*,^13^ and the haloalkaliphilic sulfur-oxidizing bacterium *Thioalkalivibrio paradoxus*.^6^ Based on the structural data of these enzymes, a pair of conserved redox-active cysteines located in the active center of FCCs on the side of the isoalloxazine ring of FAD form a disulfide bridge and are responsible for the binding of the sulfide substrate and its oxidation by the formation of transient charge transfer complex (CTC) with the FAD for the accompanied electron transfer. A similar catalytic mechanism of sulfide oxidation has also been revealed in SQR enzymes. Electrons released from sulfide are transferred from FAD to the haem c or the proximal haem c group in the monoheme or the diheme cytochrome c subunit of FCCs, respectively. Since the distance between the FAD and the haem c group is about 9 Å, it is proposed that aromatic amino acid residues of the FccB, located close to the haem group, are involved in the electron transfer^12,14^

**Figure 1:**
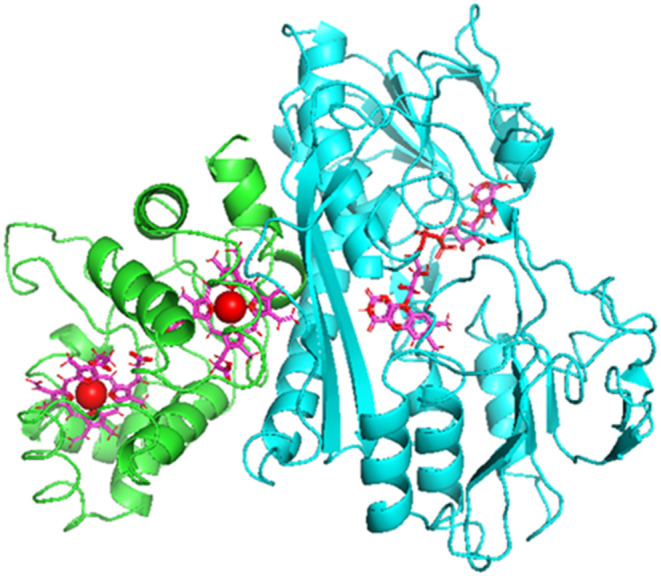
Three-dimensional structure of FccAB inferred from the homology model.

Despite a well-constructed understanding of the catalytic process of the FccAB, no experiments on the transient processes have been performed. Several ultrafast spectroscopy experiments have been conducted on other hemoproteins^15−17^ and flavoproteins,^18−22^ but very few results are available for flavohemoproteins.^23^ To the best of our knowledge, this is the first study focusing on the ultrafast dynamics of a flavocytochrome. In this article, we present the results obtained from transient absorption spectroscopy and fluorescence decay experiments to provide new insights into the transient process in FccAB. In the current studies, we take FccAB from *T. roseopersicina*. The structure of the FccAB is not yet determined. A homology model of the FccAB from *T. roseopersicina* is presented in Figure 1 to guide the results presented in this article.

## Materials and Methods

### Expression and Purification of FccAB

*T. roseopersicina* cells expressing the complex of FccA and recombinant C-terminal Strep-tag II and FLAG-tag fused FccB proteins were grown under anaerobic photoautotrophic conditions in a modified Pfenning’s medium containing 2 g L^–1^ sodium thiosulfate.^24^ Cells for protein purification were grown in 2 L Erlenmeyer flasks with a ground glass joint at 25 °C for 5 days. The medium was supplemented with kanamycin (25 μg mL^–1^), streptomycin (5 μg mL^–1^), and gentamicin (5 μg mL^–1^). Further details on the expression and purification of the protein including the structural details shall be presented in a different article.

The cultures were centrifuged at 8300*g* for 10 min at 4 °C. For isolation of the periplasmic cell fraction, harvested cells were suspended in the periplasmic buffer (150 mM NaCl, 50 mM Tris/HCl pH = 8.0, 25% sucrose, 0.1% lysozyme) and incubated at 30 °C for 30 min. To induce partial disruption of the outer membrane of cells, an equal volume of ice-cold distilled water was added, then the cells were incubated on ice for 10 min. After centrifugation of cell suspension (13,700*g*, 15 min, 4 °C), the supernatant was collected as the periplasmic fraction containing the recombinant FccAB proteins. Periplasmic fraction samples were stored at −20 °C. Recombinant FccAB protein complexes were purified by affinity chromatography via C-terminal Strep-tag II fused FccB subunit using Strep-Tactin Superflow high capacity resin (IBA Lifesciences, Cat. No.: 2–1208–002) at room temperature following the instructions of the manufacturers.

### FccAB Homology Model

Since no experimental (crystal or NMR-based) structure is available for the *T. roseopersicina* FccAB molecule, homology construction was performed for further investigations. The three-dimensional (3D) model of FccAB protein was based on the 1FCD pdb structure of *A. vinosum* flavocytochrome c,^12^ which includes two chains of the enzyme. The FccA unit consists of 200 amino acids, while the FccB chain contains 400 amino acids. The model structure should contain two cofactors (FAD and haem molecules); therefore, we used the Prime homology building package of the Schrodinger software suite (Schrödinger Release 2022–2: Prime, Schrödinger, LLC, New York, NY, 2022).^25^ The energy-based homology building method was applied, which always provides an energy-minimized single model. BLAST analysis was used for comparison of FccAB and template protein sequences. The identity rate of the FccA subunit (which contains two haem molecules with iron) was 57.56%, while the homology was 70.24%. For the FccB unit (which contains the FAD molecule), these descriptors were 68.37 and 79.77%, respectively. The quality of the model structure was checked using the PROPKA server,^26^ and both the local and overall model quality descriptors showed that the homology building process provided a reasonable structure. We would like to note that during the homology building process, the carboxyl groups of the haem molecule were kept deprotonated according to the physiological pH environment, but different protonation states (neutral and negatively charged) were also investigated in the molecular dynamics calculations.

### Time-Resolved Fluorescence

Time-resolved fluorescence measurements were carried out on FccAB produced as described in the earlier section. The details of the experimental setup combining the method of time-correlated single photon counting (TCSPC) and fluorescence upconversion can be found elsewhere.^37^ In brief, the sample, flowing through a 1 mm rectangular capillary, was excited by the frequency-doubled 750 nm pulses of an oscillator (Spectra-Physics MaiTai), emitting 375 nm∼150 fs pulses at an 80 MHz repetition rate. The fluorescence was collimated and focused by a pair of parabolic mirrors. In the upconversion arrangement, the beam of the fluorescence was mixed with the gate pulses on a BBO crystal and rotated to obtain proper phase matching. The gate was a fraction of the 750 nm fundamental beam of the oscillator, passing through an adjustable delay line and appropriate dispersion compensation. The generated sum frequency beam was introduced into a monochromator (iHR550, Horiba Jobin Yvon, France) equipped with a cooled CCD array detector (Symphony) on its first exit port. By varying the delay in the gate beam, the fluorescence decays were acquired at logarithmically spaced time points (see Figure 4) of up to 1 ns. The time resolution of this system was ∼150 fs. In the TCSPC arrangement, the beam of the fluorescence was detoured before reaching the BBO crystal by a set of flexible mirrors and was directly introduced to the monochromator. On the second exit port of the monochromator, the fluorescence was detected by a silicon avalanche photodiode (id100–50-ULN, ID Quantique, Switzerland) and acquired by a TCSPC module (SPC- 130, Becker & Hickl, Germany) with 4 ps dwell time and 40 ps time resolution. The dynamics were recorded using the upconverted wavelength of ∼300 nm (1/300 = 1/750 + 1/500 using a BBO crystal for sum frequency generation at an appropriate phase matching angle) corresponding to 500 nm emission wavelength. All of the fluorescence decay results presented in the article were obtained at the emission wavelength of 500 nm where the emission was observed to be maximum. Reconvolution fits with exponential functions were performed to retrieve the fluorescence lifetimes (Figure S1 and Table S1). Exponential component analysis of fluorescence decay curves was performed using the software FAST (Edinburgh Instruments Ltd.).

### Transient Absorption Spectroscopy

The broadband transient absorption setup has been described in detail elsewhere.^27^ It is based on a pair of synchronized titanium:sapphire amplifiers (Femtopower and Solstice, both manufactured by Spectra-Physics company) synchronized by sharing the oscillator (Element, Spectra-Physics). For probe pulses, Femtopower output was focused into hollow core fiber filled with argon gas generating white light in the spectral range 290–1000 nm. Pump pulses were generated from the Solstice lasers by wavelength conversion in commercial OPA system TOPAS (Light Conversion). They were prepared at 360, 410, and 560 nm. The pump and probe spot sizes at the sample were 100 and 50 μm, respectively. The pump fluence used for the experiments reported in this article were 2.4, 1.6, and 3.7 mJ/cm^2^ respectively. The fluence used were confirmed to avoid any nonlinear excitation (Figure S2).^15^ The spectrally dispersed probe was recorded with a CCD camera (1034 pixels, Entwicklungsbuero Stresing) operating at 1 kHz in shot to shot regime. Probe white light fluctuation was corrected by measuring reference spectra with the same sampling and resolution as signal spectra. The pump–probe cross-correlation was about 100 fs over the full spectral range and mutual polarization set to magic angle 54.7° to eliminate rotational and anisotropic effects. The transient absorption spectra were corrected for the chirp of the supercontinuum and for the solvent signal.^27^ The spectra were recorded in 20 fs steps in the IRF window and logarithmic sampling at long delays. For all of the measurements presented, the sample was placed in a quartz cuvette of path length ∼1 mm. The sample volume in the cuvette was ∼150 μL.

### Analysis of Transient Absorption Spectroscopy

Transient absorbance changes from femtoseconds to nanoseconds in the spectral region from 290 to 1000 nm (Figure S3A) were subjected to lifetime distribution analysis (LDA) by the maximum entropy method^28−30^ as previously described.^31,32^ Prior to analysis, the data were logarithmically averaged. For LDA, the lifetimes were fixed and logarithmically distributed from 2 × 10^–14^ s (the first experimental time delay point) to 10^–9^ s with 20 points per decade. The wavelength- and time-dependent amplitudes were the only fitting parameters. The l-curve criterion was used to select the optimal regularization parameter. From the two-dimensional (2D) lifetime density maps (Figure S3B), the lifetime-dependent average dynamical content *D* (Figure 6A) was computed as the square root of the summation over the squared amplitudes.^33,34^ Events under 0.15 ps, which may be attributed to the coherent artifact and/or cross-phase modulation,^35^ were not analyzed further. The peak centers of the *D* lifetime distribution were retrieved (Table 1). The decay-associated difference spectra (DADS, Figure 6B) were extracted from the 2D lifetime distribution upon integration of the transient spectra in the corresponding time range. Original and fitted time traces are shown in Figure S4A

**Table 1:** Summary of the Major Dynamical Process Observed in the Transient Absorption Spectra of FccAB Obtained by Lifetime Distribution Analysis

	λ_actinic_ (nm)			
components	360	400	560	assignment
**τ**_**①**_ (ps)	0.4	0.5	0.3	internal conversion
τ_②_ (ps)	4.3	4.6	4.3	vibrational cooling

As an alternative to LDA and to exclude the possibility of data misinterpretation due to not accounting for the impulse response function (IRF), we have performed multiexponential fitting i.e., global kinetic analysis (GKA) using the software described in Lórenz-Fonfría et al.^36^ The IRF was modeled as a Gaussian distribution and fitted (Table S2) and deconvolved together with the rest of components (Table S3). Original and fitted time traces are shown in Figure S4B. The optimal number of components was determined by singular value decomposition (SVD) analysis. The fraction of intermediate species as a function of pump–probe time delay (Figure S5A) and the evolution-associated difference spectra (EADS, Figure S5B) were extracted from the fits assuming a sequential irreversible model; 1 → 2 → *n* (where *n* is the total number of components). Given the width of the IRF (up to 0.15 ps), events under 0.15 ps were disregarded.

## Results and Discussion

### Structural Homology Model of *T. roseopersicina* FccAB Dimer

**Figure 2:**
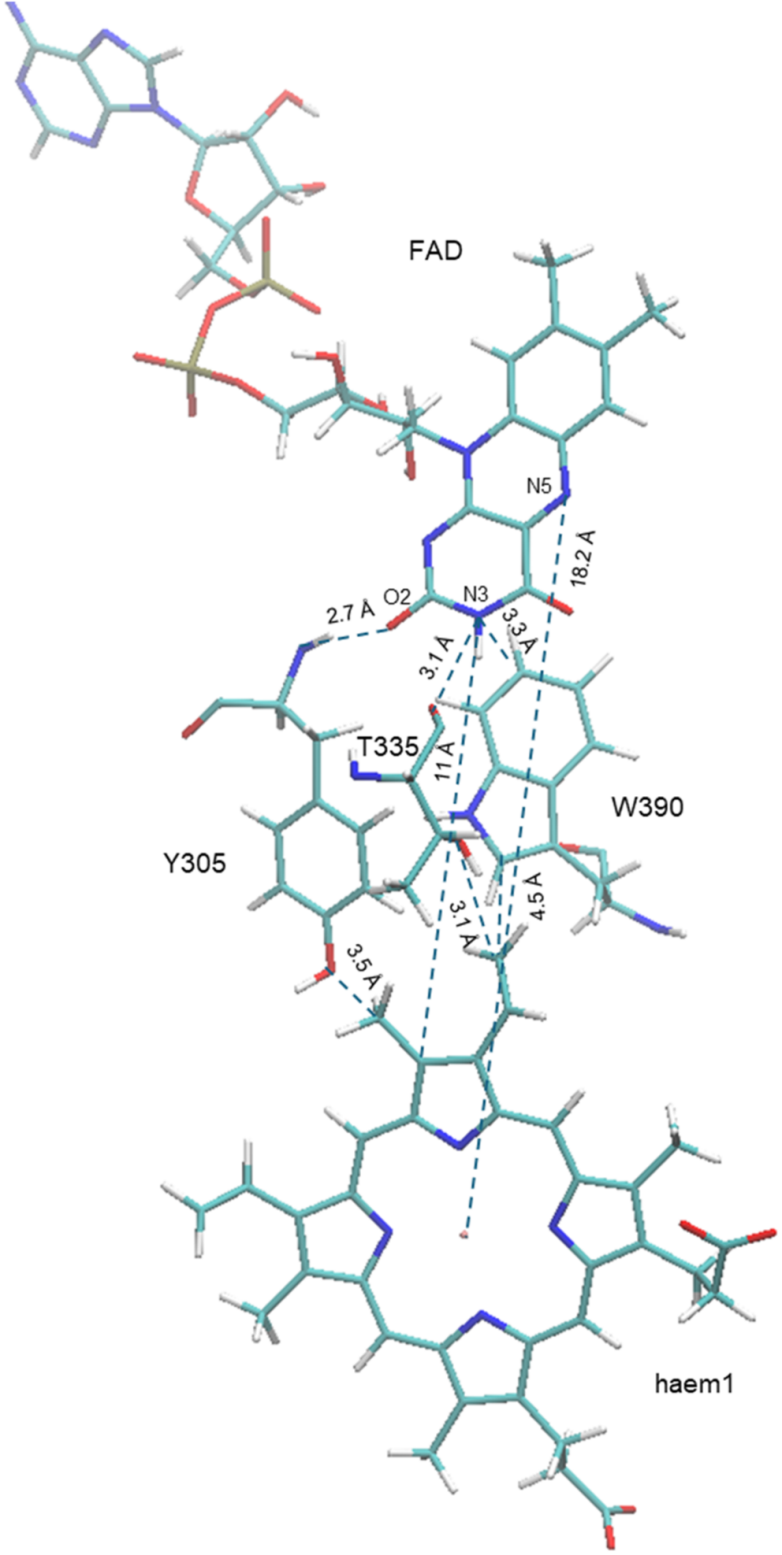
Arrangement of FAD and haem1 prosthetic groups in the structural model of *T. roseopersicina* FccAB and potential pathways of electron flow between the flavin and haem. Distance between different groups of the residues in the electron transport pathways is indicated by broken lines.

In the absence of a crystal structure for FccAB of *T. roseopersicina*, an energy-minimized homology structural model was constructed for this flavocytochrome c enzyme. Details of the model building are presented in the Materials and Methods Section. The FAD and haem cofactors were also incorporated in the large and small subunits of the model of the dimeric protein complex, respectively. The structure derived from the homology model is shown in Figure 1. The shortest distance between the isoalloxazine ring of the flavin and pyrrole rings of the haem molecules is 11 Å. The iron in the proximal haem1 prosthetic group is 18.2 Å from the N-5 atom in the flavin. The two iron atoms in the haem1 and haem2 moieties are separated by 19 Å, while the closest distance between the edges of the porphyrin rings is 12.6 Å. Tyr305, Thr335, and Trp390 amino acids in FccB lie between the FAD and the interface of the subunits (Figure 2). These residues correspond to Tyr306, Thr366, and Trp336 in *A. vinosum* FccB, respectively, and their side chains provide potential pathways for electron flow from flavin to haem in all flavocytochrome c enzyme complexes.^6,12,14^ Proposed π–π interactions of aromatic side chains and hydrogen bond of the residues may form electron transport chains between the prosthetic groups. Distances between the amino acids and different groups of the FAD and haem1 molecules are about 3–4 Å in the *T. roseopersicina* FccAB proteins (Figure 2).

### Steady-State Spectroscopy of Oxidized and Reduced Forms of FccAB

**Figure 3:**
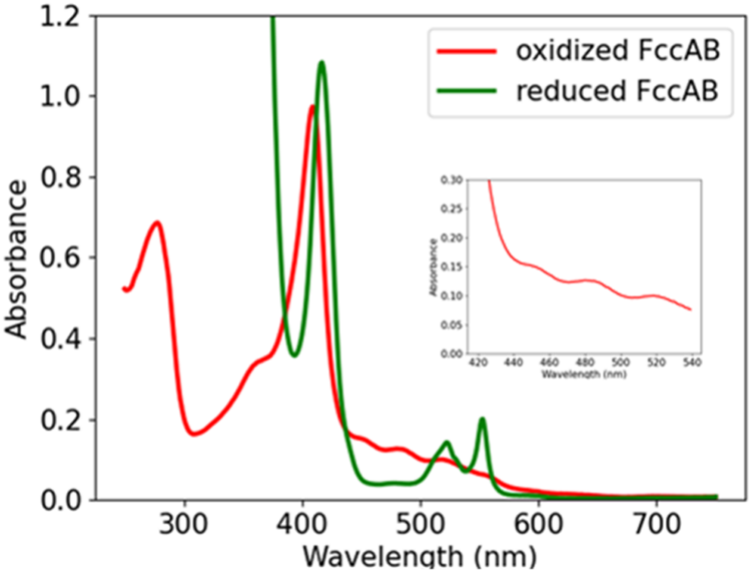
Steady-state ultraviolet/visible absorbance spectra of FccAB. The inset shows the magnified view of the peaks present at ∼450 and 480 nm in the oxidized state.

In order to distinguish the oxidized and reduced state of FccAB, steady-state absorption spectrum is presented in Figure 3. The red line shows the absorption spectrum of the freshly prepared FccAB. The iron atoms of heme are assumed to be completely oxidized under these conditions. The spectrum shows mainly four bands in the range of 300–500 nm: the Soret band of the heme chromophore at 409 nm, two bands at ∼450 and ∼484 nm that can be attributed to the FAD moiety in the protein, and a broad peak ranging from 500 to 570 nm most likely representing the Q-bands of heme. The sample was further reduced by dithionite, and the spectrum of the reduced FccAB is shown in Figure 3. The reduced spectrum shows the red shift of the Soret band by 8 nm, and the Q-band peaks are amplified showing two distinct peaks at 522 and 552 nm. In the reduced form, the bleaching of the peaks at ∼450 and 480 nm is consistent with the previous observations in the flavoproteins.^38^ These observations underline the significant differences in the electronic environments of the heme and FAD moieties between the oxidized and reduced states of FccAB. The red shift in the Soret band and the amplification of the Q-bands upon reduction highlight the dynamic nature of these components. However, while steady-state absorption spectroscopy provides a snapshot of these static electronic states, it does not capture the rapid processes and transitions that occur on ultrafast time scales within the enzyme. To fully understand the efficiency and mechanism of electron transfer and other dynamic processes, further investigation into the temporal aspects of these transitions is essential.

### Time-Resolved Fluorescence of FccAB

To analyze the dynamic behavior of FccAB, we employed time-resolved fluorescence and measured the rapid decay of fluorescence from the FAD moiety, accounting for the fact that heme is poorly fluorescent.^39,40^ This approach allows us to capture the fleeting moments immediately following photoexcitation, providing insights into the speed and efficiency of internal electron transfer processes. By examining these ultrafast dynamics, we can elucidate the roles and interactions of the enzyme components during its catalytic cycle, thereby addressing the limitations of steady-state spectroscopy and offering a detailed understanding of the dynamical aspects of FccAB functionality.

**Figure 4:**
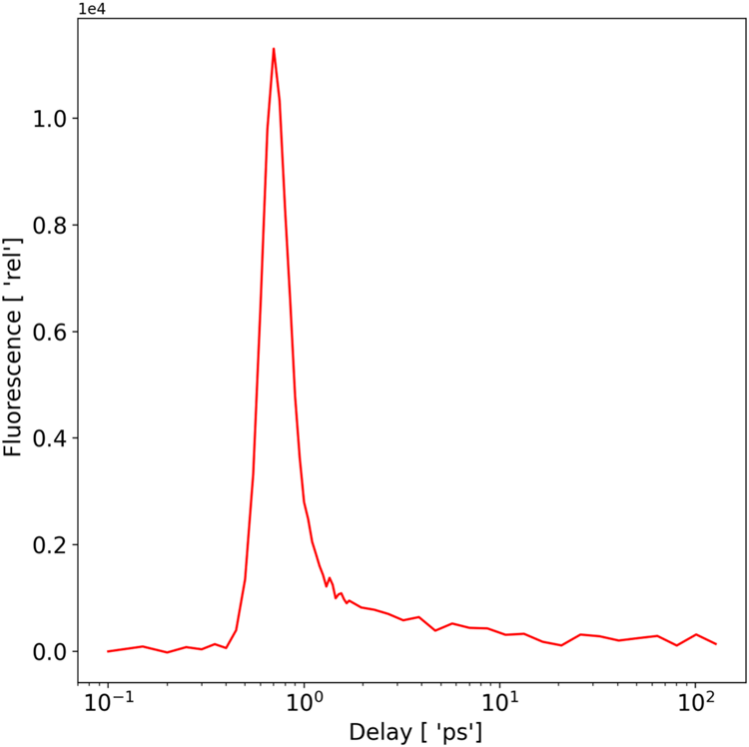
Fluorescence decay at magic angle measured from the FccAB at 375 nm excitation and 500 nm emission wavelength.

In Figure 4, we present the fluorescence dynamics of FccAB upon 375 nm excitation and 500 nm emission obtained at the magic angle. We believe that the FAD is the dominant contributor to our fluorescence decay curve because (i) heme is poorly fluorescent (quantum yield less than 10^–6^,^41−43^ compared to the 10^–2^ quantum yield of FAD)^44−46^ and (ii) heme fluorescence emission upon excitation at 375 nm is centered at 430 nm (520 nm in the case of FAD).^47,48^The fluorescence decay has been fitted with a double exponential. However, only the 0.6 ps lifetime component may correspond to a truly dynamical event, while the 0.1 ps lifetime component lies within the time scale of the IRF (Figure S1 and Table S1). The very fast (majority happening in less than 10 ps) decay of the FAD fluorescence located at the center of the FccAB molecule suggests that the close proximity between FAD and heme provides a pathway for quenching by intramolecular electron transfer.

If our interpretation is correct, the FAD molecule has a crucial role in the electronic energy transfer within the FccAB.

While this single measurement does not provide in-depth understanding of the underlying processes, it is consistent with an electron transfer pathway happening in FccAB protein on a time scale of less than 10 ps, which indicates the efficiency and promptness of these interactions. While these results provide insights into the dynamics of FccAB, they also raise further questions about the detailed mechanisms and specific pathways of energy transfer and relaxation processes within the enzyme.^49−52^

### Transient Absorption Spectroscopy

To further elucidate the underlying photodynamic processes within FccAB, we conducted transient absorption spectroscopy experiments with excitations at three different wavelengths (360, 400, and 560 nm). This technique allows us to capture the evolution of excited-state absorbance spectra over the relevant picosecond time domain, capturing dynamics at play. By analyzing the time-resolved absorbance spectra, we can identify and characterize the specific events and transitions occurring within the enzyme, offering a view of its ultrafast photodynamics and contributing to a fuller understanding of its catalytic mechanism. A power scan of the excitation for all three pump wavelengths is given in Figure S2 confirming that experiments were performed in the linear excitation regime.

**Figure 5:**
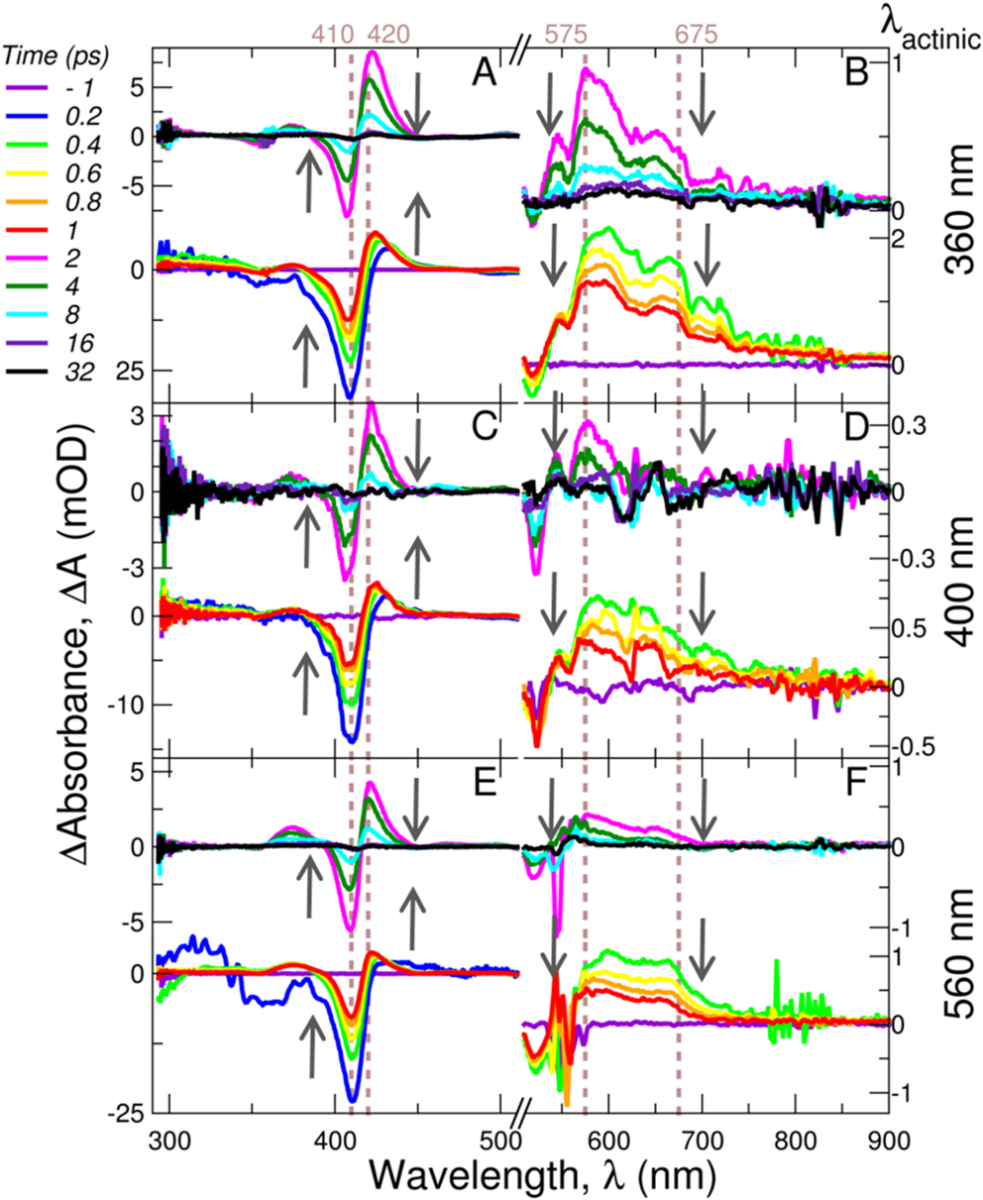
Transient absorption spectra at selected time delays upon excitation with three different pump wavelengths shown on the right. Spectra have been split into two regions: under 500 nm (left) and above 500 nm (right): (A, B) for pump at 360 nm; (C, D) for pump at 400 nm, and (E, F) for pump at 560 nm. Within each panel, bottom spectra show subps delays and top spectra show multips delays. Vertical dotted lines are intended to guide the eye. Vertical arrows indicate the temporal evolution of the negative bands (ground state bleach) at ∼410 nm, and positive bands at ∼420, ∼575, and ∼675 nm (excited-state absorption).

**Figure 6:**
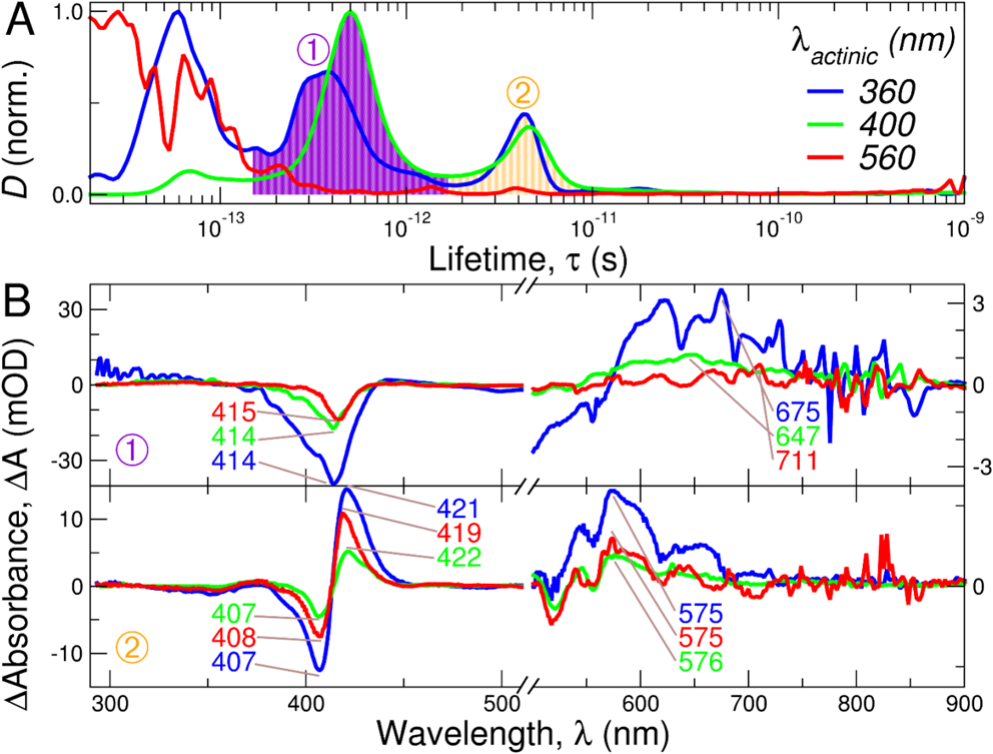
Lifetime distribution analysis. (A) Normalized average dynamical content (*D*) as a function of lifetime (τ). Two main components, labeled ① and ② were found (sub-0.1 ps events were disregarded). (B) Decay-associated difference spectra (DADS) obtained by integration of the transient spectra in the time range indicated by the shaded areas of panel (A). Due to the large difference in intensities, the spectra were separated into two ranges: below 500 nm (left) and above 500 nm (right). The position of the main differential absorption bands (in nm) is indicated.

Figure 5 displays the transient absorption spectra of FccAB for all three pump wavelengths at selected time delays. Figure 5(A,C,E) focuses on the 300–500 nm region, and Figure 5(B,D,F) focuses on the 500–900 nm region. The spectral evolutions of FccAB upon excitation with three different wavelengths (360, 400, or 560 nm actinic pumps) are qualitatively similar (Figure 5), showing negative bands (ground state bleach) at ∼410 and ∼525 nm and positive bands (excited-state absorption) at ∼420, ∼575, and ∼675 nm. However, upon close inspection of the plots, small shifts in band positions depending on the pump wavelength can be seen (Figure 5, dotted lines). To gain further insight, we subjected the time-resolved absorbance spectra of FccAB to lifetime distribution analysis (Figure S3b). The derived dynamical contents (*D*) and decay-associated difference spectra (DADS) are shown in Figure 6A,6B, respectively. The *D* lifetime distribution shows two major events occurring at time delays of ∼0.4 and ∼4 ps (Table 1). Similar relaxation times have been previously reported for cytochrome c upon ultraviolet (UV) (266 nm) and visible (403 and 530 nm) excitation.^53,54^ The fastest event is characterized by a narrow negative band at around ∼415 nm, and a broad positive band at ∼700 nm with fine structure.

The subpicosecond decay of the ground state bleach (GSB) band has been previously ascribed to the internal conversion of excited heme to the electronic ground state.^53,55^ The slowest event (∼4 ps) is characterized by a negative/positive pair at 410/420 nm. It has been previously interpreted as the vibrational cooling of a “hot” ground state due to its similarity to hot stationary differential absorption spectra.^53,55^

We also subjected our time-resolved absorption data to global kinetic analysis (GKA) using multiexponential fitting and deconvolution of IRF. The results of GKA (Figure S5) are in agreement with the results arising from LDA, both strongly supporting the presence of two major dynamical events in FccAB regardless of the excitation pump (360, 400, or 560 nm): subpicosecond (∼0.4 ps) internal conversion, and picosecond (∼4 ps) vibrational cooling. However, the relative abundance (spectral amplitudes) of such events for all of the excitations used in this experiment follows the order: 360 > 400 ∼560 nm.

Interestingly, while the sub-500 nm transient spectra (Figure 5, left) clearly resemble those of heme and heme proteins, the spectra at wavelengths longer than 500 nm (Figure 5, right) do not seem to recapitulate those of flavoproteins.^32,56−58^ Picosecond time scale spectra of flavins and flavoproteins generally display stimulated emission (SE) at ∼550 nm and excited-state absorption at ∼800 nm that are characteristic of the excited singlet states.^32,56^ Upon intersystem crossing in the nanosecond time regime, the transient spectrum of the excited triplet states typically peaks at ∼700 nm^32,57,58^

**Figure 7:**
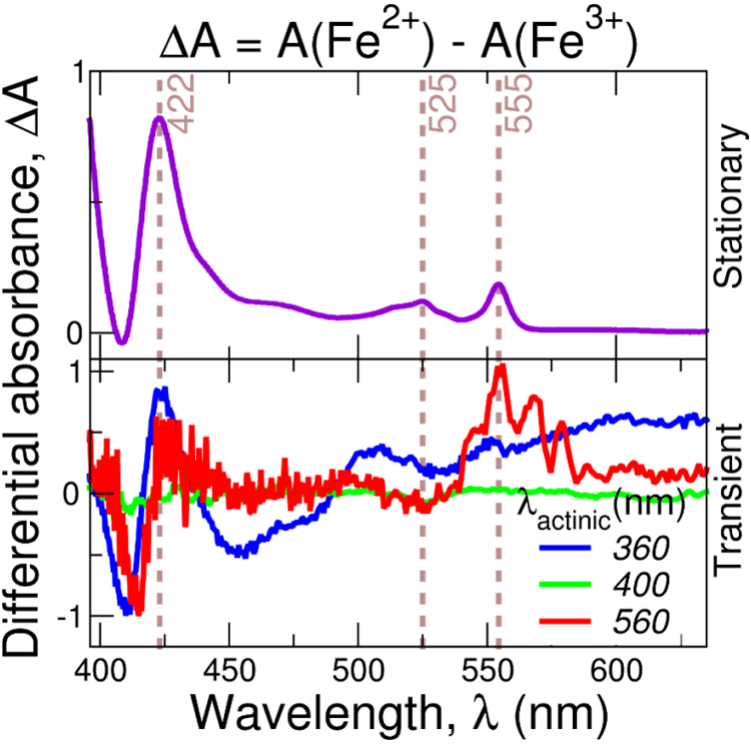
Differential absorption spectra (Δ*A*) of flavocytochrome c. (A) Steady-state difference spectra between dithionite-reduced (Fe^2+^) and oxidized (Fe^3+^) FccAB. (B) Transient spectra of FccAB averaged over a pump–probe delay between 20 and 60 ps. The wavelengths of the actinic pump are indicated.

Chemical reduction of flavocytochrome c gives rise to three characteristic bands at 422, 525, and 555 nm (Figure 7A). The relative position of these bands is similar to that of reduced cytochrome c, suggesting that they arise mainly from the heme chromophore with minor contributions from FAD. Some of the bands in the spectra of Figure 7A appear to be present in DADS of the second kinetic component with a lifetime of ∼4 ps (Figure 6B). Next, we looked at the transient spectra after 20 ps (Figure 7B) when the vibrational cooling should be completed and the signals from the reduced form should be seen more clearly. The 422 nm band is present upon photoexcitation by all three wavelengths used (360, 400, and 560 nm). Thus, we found evidence of heme photoreduction happening a few picoseconds after UV/vis excitation. However, whether photoreduction occurs only in “cold” heme or it already starts during the cooling process is difficult to determine. In any case, it appears that the ultrafast photooxidation of the FAD is followed by the photoreduction of the heme group in the FccAB.

The transient absorption spectroscopy results reveal picosecond scale spatial-temporal insights into the ultrafast photodynamic processes within FccAB. The analysis of the spectral evolution upon excitation at different wavelengths highlights two major dynamic events: a subpicosecond internal conversion and a few-picoseconds vibrational cooling. These observations align well with previously reported relaxation times for similar heme proteins^59,60^ and provide a detailed understanding of the rapid electronic transitions and energy dissipation pathways. Despite the comprehensive nature of our findings, we sought further validation of the cooling processes as necessary to fully corroborate the interpretations derived from the transient absorption data.

**Figure 8:**
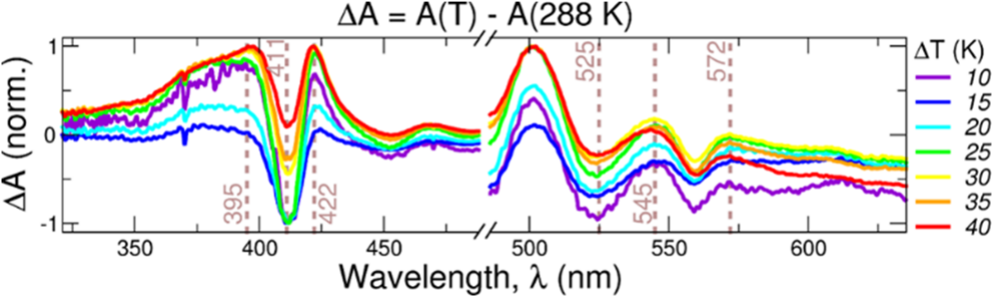
Temperature-dependent stationary differential absorption spectra Δ*A* at a given temperature (*T*) relative to the spectrum at *T* = 288 K, Δ*A* = *A*(*T*) – A(288 K), for oxidized flavocytochrome c. For small differences in temperature (Δ*T* < 40 K), the positions of the main absorption bands (indicated with dashed lines) are similar to the “hot” transient absorption spectra shown in Figure S3B.

As mentioned before, it has been hypothesized that until the cooling is complete, the hot transient spectra should resemble the hot stationary spectra. In order to confirm such a hypothesis, we monitored the UV/vis absorption spectrum of oxidized FccAB in the temperature range 288–353 K. The resulting difference absorption spectra are shown in Figure 8. Both the DADS of the component decaying in ∼4 ps and the steady-state spectra show a negative band at ∼410 nm and a positive band at ∼420 nm. Above 500 nm, the spectra are qualitatively more different, but some common features can be found like the negative/positive/positive bands at 525, 550, and 575 nm, respectively. Overall, we conclude that the “hot” ground state interpretation of the second main dynamical event is plausible.

## Conclusions

The comprehensive study of flavocytochrome c sulfide dehydrogenase (FccAB) utilized steady-state absorption spectroscopy, time-resolved fluorescence kinetics, transient absorption spectroscopy, and temperature-dependent steady-state spectroscopy to elucidate its ultrafast dynamics and energy transfer mechanisms. Steady-state spectroscopy distinguished between oxidized and reduced states, highlighting significant spectral shifts and amplification of characteristic bands upon reduction. These findings underscored the distinct electronic environments of the heme and FAD moieties. Time-resolved fluorescence kinetics revealed rapid FAD fluorescence decay within less than 10 ps, suggesting efficient internal electron transfer. This highlighted the crucial role of FAD cofactor in the enzyme’s energy transfer dynamics. Transient absorption spectroscopy further detailed the photodynamic processes, identifying subpicosecond internal conversion and vibrational cooling events. These observations aligned with known relaxation times for other heme proteins, validating the proposed mechanisms of electronic transitions. The dynamics observed in FccAB in this report is also similar to other flavoenzymes previously reported.^61^ Temperature-dependent steady-state spectroscopy confirmed that the cooling dynamics corresponded to ″hot″ stationary states by comparing UV/vis spectra at various temperatures. This correlation reinforced the vibrational cooling interpretation. Integrating these techniques provided a comprehensive understanding of FccAB dynamic behavior, elucidating rapid (picosecond time scale) intramolecular electron transfer from FAD to heme and energy dissipation processes, and contributing significantly to the knowledge of its catalytic mechanisms and functional dynamics.

**Figure 9:**
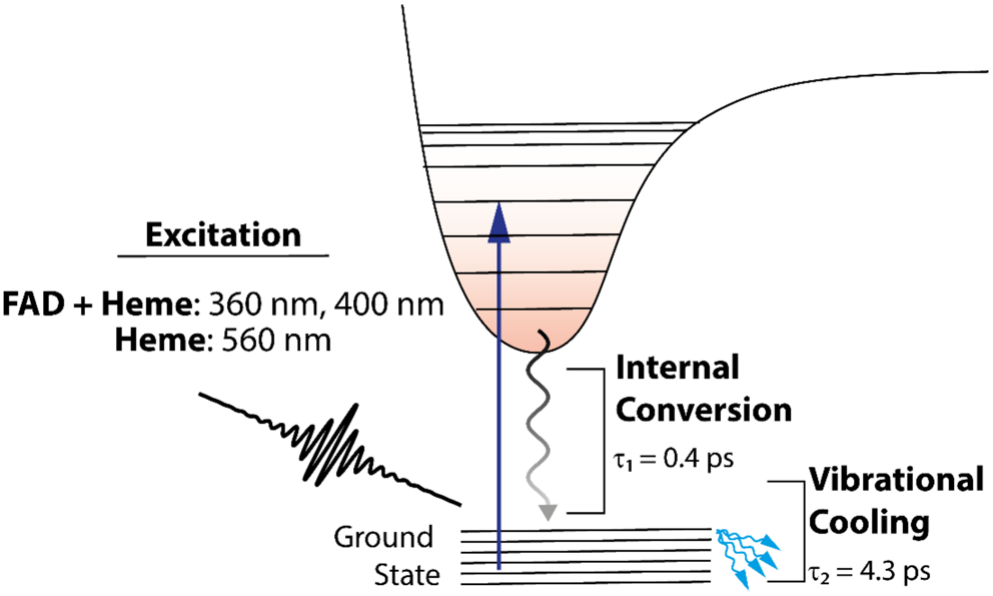
Schematic of the summary of the ultrafast processes from the experiment. The schematic shows that excitation at three different excitation wavelengths has been used and two major processes have been observed for each excitation. The electric field used in the scheme is to represent the Gaussian pulse intensity.

A summary of the process observed in the experiment is presented in Figure 9.

## Data Availability

All “chirp-corrected” and “derived” (arising from lifetime distribution analysis and global kinetic analysis) transient absorption data sets have been deposited in Zenodo (https://doi.org/10.5281/zenodo.14030391)

## Supporting Information Available

Reconvolution fits of the fluorescence decay of FccAB measured at the magic angle (data) with a single-exponential function (fit 1 component) or a double-exponential function (fit 2 components); fluence-dependent difference absorbance of the positive peak ∼420 nm for excitation wavelengths 400, 360, and 560 nm; raw (chirp-corrected) and transformed dataset; and results from global kinetic analysis of the raw (chirp-corrected) transient absorption spectra of FccAB (PDF)
