# Supplementary material for: Ultrafast Dynamics in Flavocytochrome C by Using Transient Absorption and Femtosecond Fluorescence Lifetime Spectroscopy
Source: J Phys Chem B. 2025 Apr 8;129(15):3731–9. doi: 10.1021/acs.jpcb.4c05496 (PMC12010331; doi:10.1021/acs.jpcb.4c05496)
Supplement: Supplementary file 1 — jp4c05496_si_001.pdf [file jp4c05496_si_001.pdf]

Ultrafast Dynamics in Flavocytochrome C by using Transient Absorption and Femtosecond Fluorescence Lifetime Spectroscopy

Krishna P. Khakurel,[a] Gustavo Fuertes, [b] Aron Sipos, [c] Gábor Paragi, [d,e,f] Jakub Dostal, [a] Miroslav Kloz, [a] Gabriel Žoldák, [g] Jakob Andreasson, [a] and András Tóth* [c,h].*

[a] *Extreme Light Infrastructure ERIC, CZ-25241 Dolni Brezany, Czech Republic.*

[b] *Institute of Biotechnology of the Czech Academy of Sciences, CZ-25250 Vestec, Czech Republic.*

[c] *Institute of Biophysics, HUN-REN Biological Research Centre, H-6726 Szeged, Hungary.*

[d] *Institute of Physics, University of Pécs, Ifjúság útja 6., H-7624, Pécs, Hungary*

[e] *Department of Theoretical Physics, University of Szeged, Tisza Lajos krt. 84-86, H-6720 Szeged, Hungary*

[f] *Department of Medicinal Chemistry, University of Szeged, Dóm tér 8, H-6720 Szeged, Hungary*

[g] *Faculty of Science, Pavol Jozef Šafárik University in Košice, Park Angelinum 19, 040 01 Košice, Slovakia*

[h] Department of Biotechnology and Microbiology, University of Szeged, H-6726 Szeged, Hungary

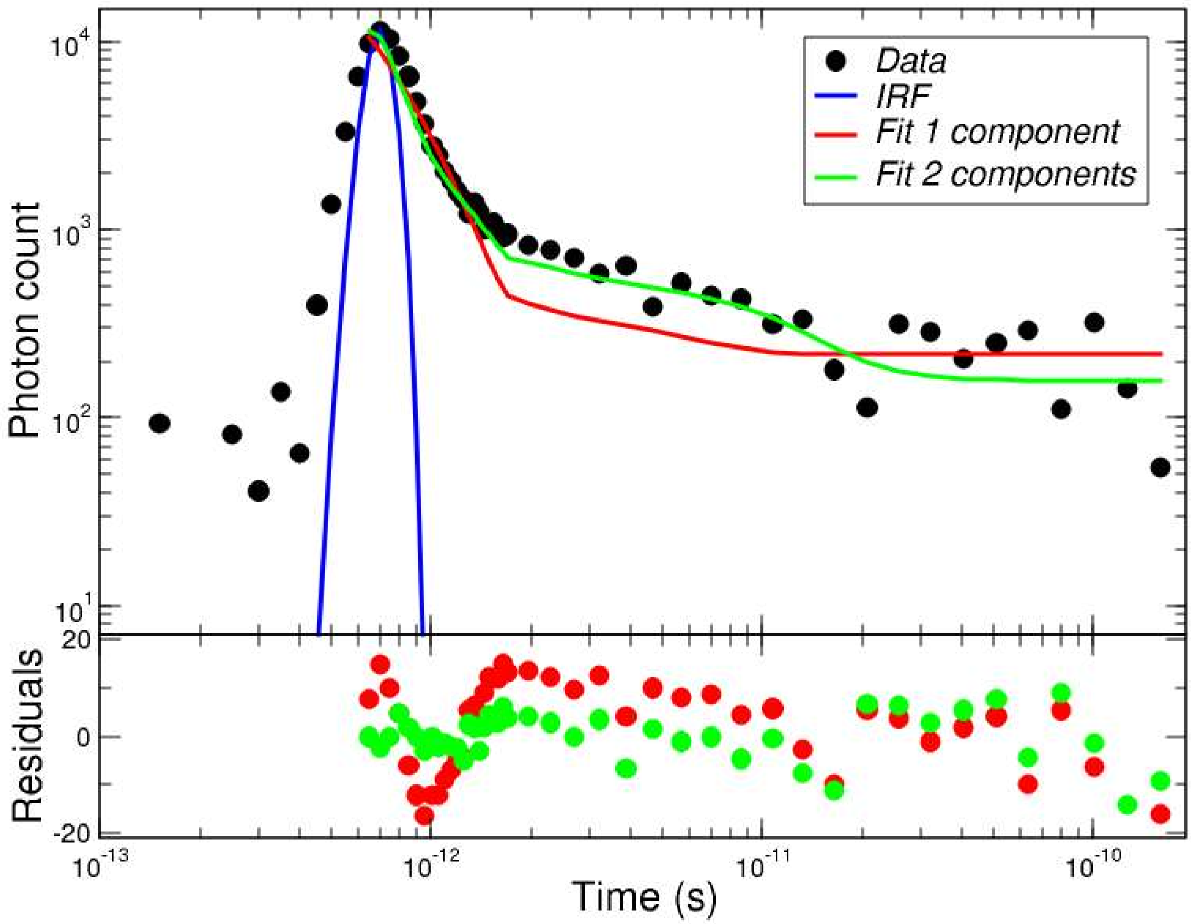

Fig. S1. Reconvolution fits of the fluorescence decay of FccAB measured at the magic angle (data) with a single exponential function (fit 1 component) or a double exponential function (fit 2 components). The IRF was modeled as a Gaussian with mean = 0.7 ps and FWHM = 150 ps). The fitted lifetimes are listed in Table S1.

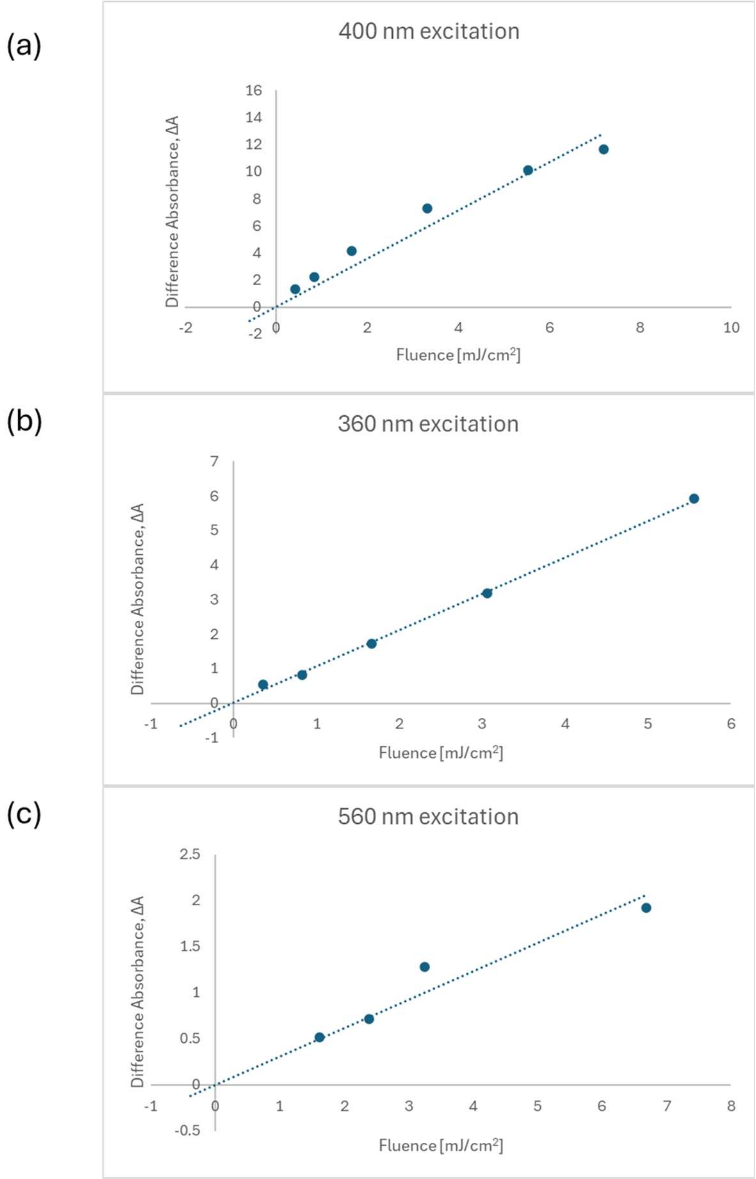

Fig S2. The fluence dependent difference absorbance of the positive peak ~ 420 nm for excitation wavelengths (a) 400 nm (b) 360 nm and (c) 560 nm.

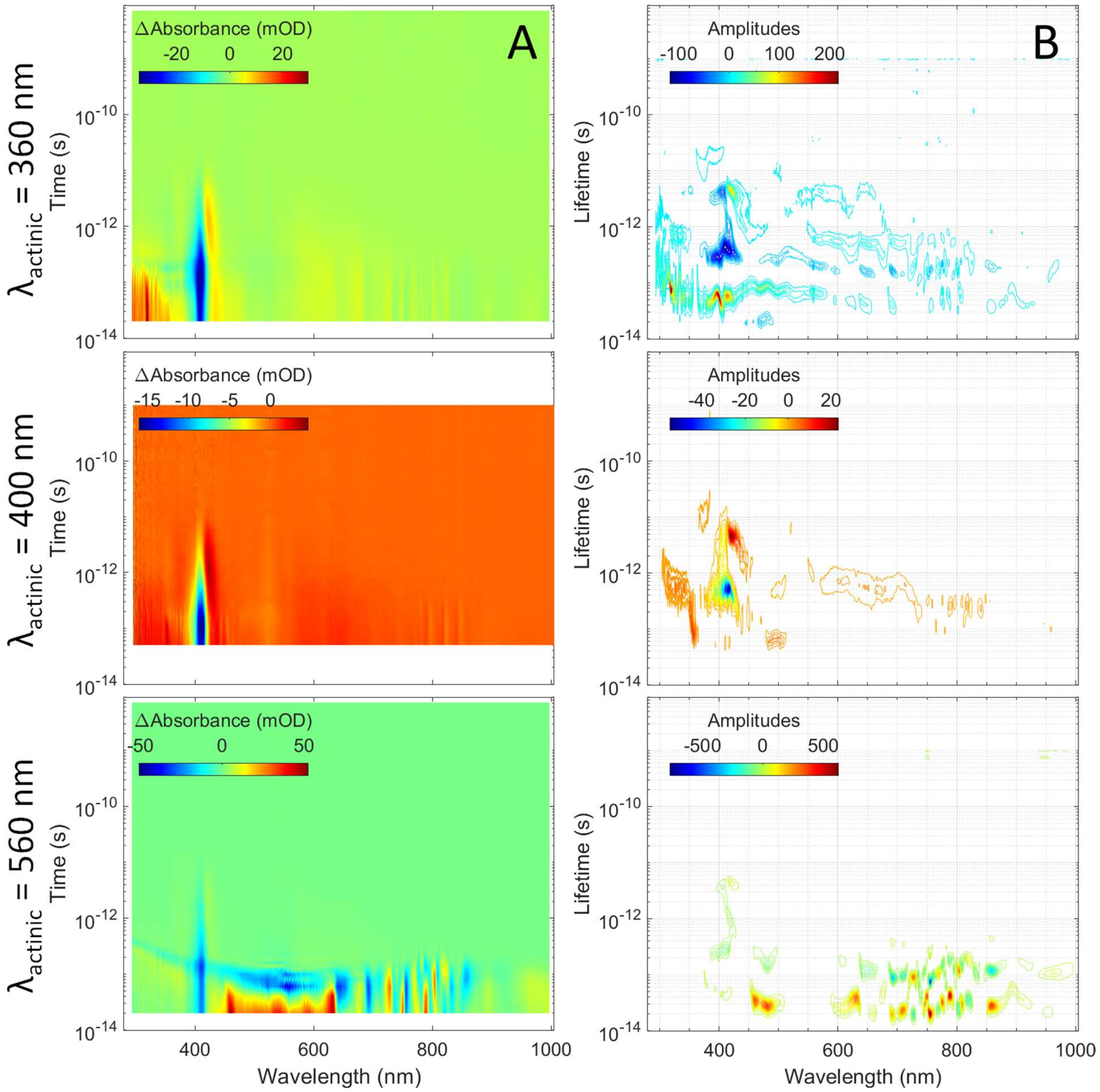

Fig S3. Raw (chirp-corrected) and transformed datasets. (A) Experimental 2D contour plots of differential absorbance (in mOD) as a function of pump-probe delay and wavelength. (B) Computed 2D contour plots of fitted amplitudes as a function of lifetime and wavelength using the inverse Laplace transform and the maximum entropy method.

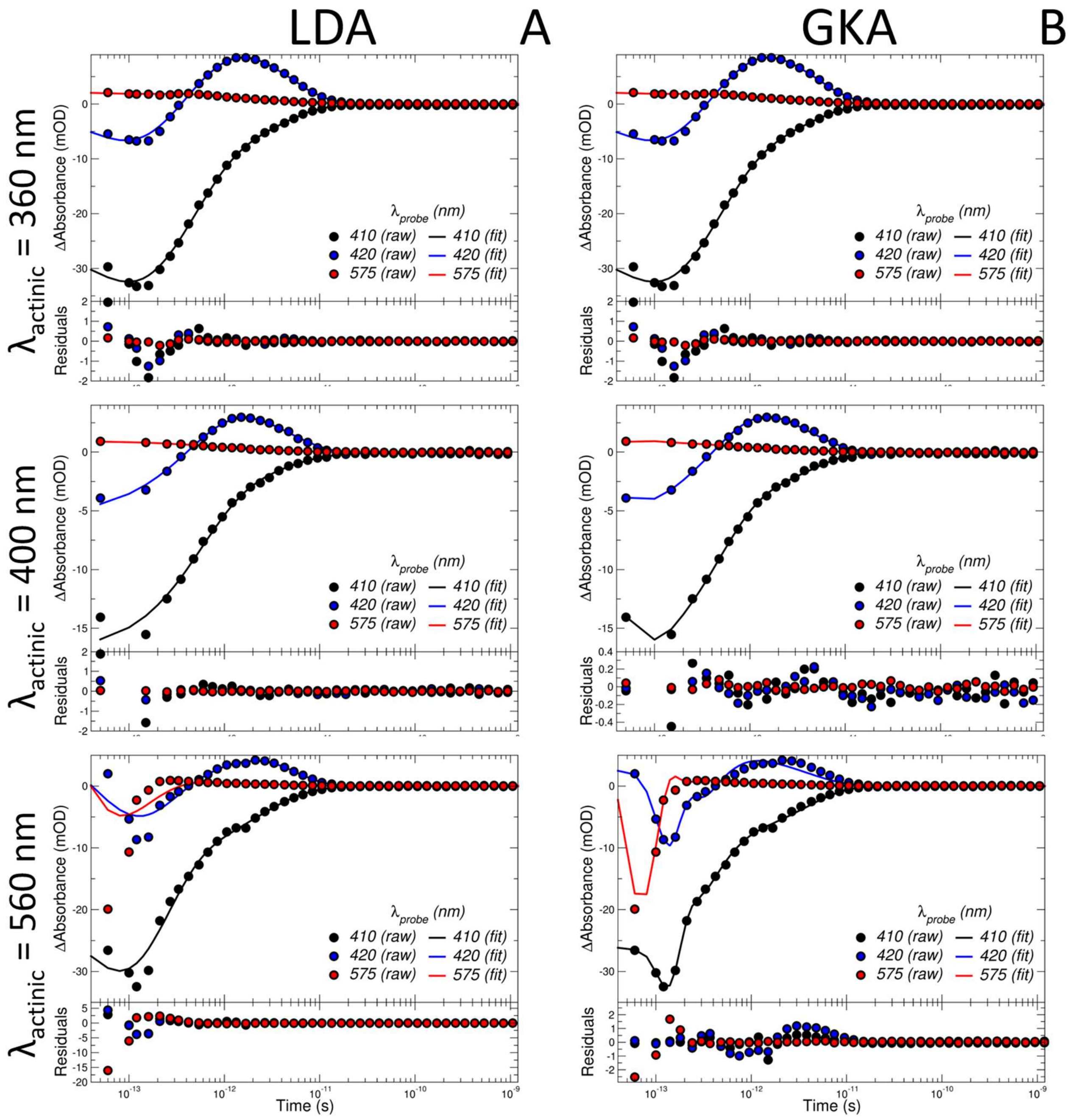

Fig S4. Raw (chirp-corrected) and fitted transient absorption time traces at three selected probe wavelengths (410, 420 and 575 nm). (A) Lifetime distribution analysis (LDA). (B) Global kinetic analysis (GKA).

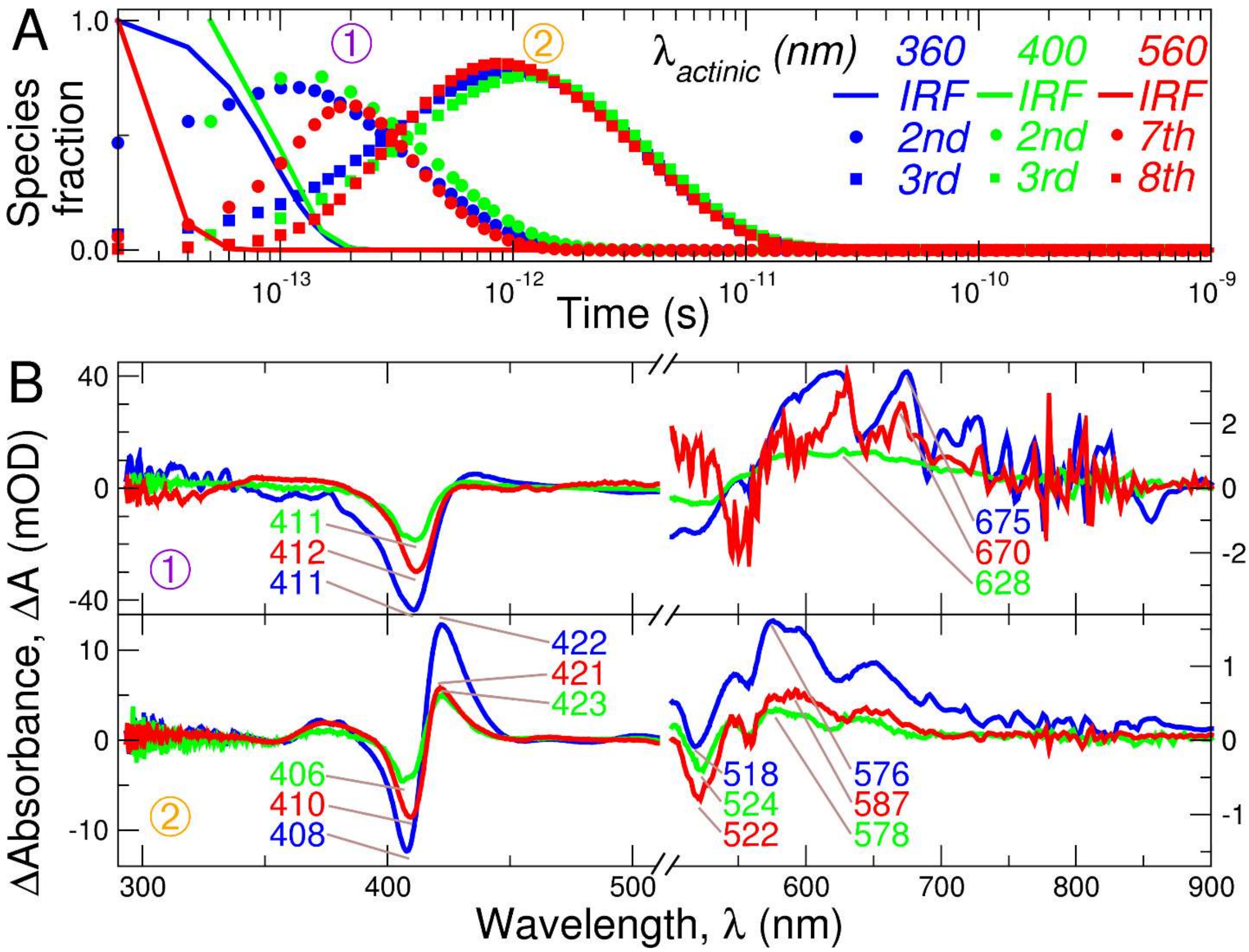

Fig. S5. Results from global kinetic analysis of the raw (chirp-corrected) transient absorption spectra of FccAB. (A) Intermediate fraction as a function of pump-probe time delay assuming a sequential model. For the sake of clarity, only the last two intermediates are drawn, labeled ① and ②. The fitted impulse response function (IRF), modeled as a Gaussian, is also shown in the graph. (B) Evolution-associated difference spectra (EADS) of the two intermediate species shown in panel A. Due to the large difference in intensities, the spectra were separated in two ranges: below 500 nm (left) and above 500 nm (right). The position of the main differential absorption bands (in nm) is indicated.

Table S1. Results of fitting the fluorescence decay of FccAB with an exponential function (reconvolution).

	**Single exponential**		**Double exponential**	
**Component**	B_i	τ_i *(ps)*	B_i	τ_i *(ps)*
1	**0.831**	**0.3**	**1.958**	**0.1**
2	**-**	**-**	**0.125**	**0.6**

B_i are the amplitudes (pre-exponential factors) and τ_i are the lifetimes.

Table S2. Results of fitting the instrumental impulse response function (IRF) with a Gaussian distribution.

		**Position (ps)**	**FWHM (ps)**
**$\lambda_{actinic}$ (nm)**	**360**	**0.006**	**0.15**
	**400**	**0.025**	**0.13**
	**560**	**0.001**	**0.10**

Table S3. A summary of the major dynamical process observed in the transient absorption spectra of FccAB obtained by global kinetic analysis.

**Components**	**$\lambda_{actinic}$ (nm)**			**Assignment**
	**360**	**400**	**560**	
τ①* *(ps)	**0.4**	**0.4**	**0.3**	**Internal conversion**
τ②* *(ps)	**3.8**	**4.1**	**3.7**	**Vibrational cooling**
